# Physiological and Biomechanical Responses of Highly Trained Distance Runners to Lower-Body Positive Pressure Treadmill Running

**DOI:** 10.1186/s40798-017-0108-x

**Published:** 2017-11-21

**Authors:** Kyle R. Barnes, Jessica N. Janecke

**Affiliations:** 1 Department of Movement Science, Grand Valley State University, 1 Campus Drive, Allendale, MI 49401 USA; 2 Office of Undergraduate Research and Scholarship, Grand Valley State University, 1 Campus Drive, Allendale, MI 49401 USA

**Keywords:** AlterG, Lower-body positive pressure, Body weight support, Anti-gravity, Running, Stride characteristics, Physiological characteristics, Metabolic demand, Oxygen demand, Oxygen cost

## Abstract

**Background:**

As a way to train at faster running speeds, add training volume, prevent injury, or rehabilitate after an injury, lower-body positive pressure treadmills (LBPPT) have become increasingly commonplace among athletes. However, there are conflicting evidence and a paucity of data describing the physiological and biomechanical responses to LBPPT running in highly trained or elite caliber runners at the running speeds they habitually train at, which are considerably faster than those of recreational runners. Furthermore, data is lacking regarding female runners’ responses to LBPPT running. Therefore, this study was designed to evaluate the physiological and biomechanical responses to LBPPT running in highly trained male and female distance runners.

**Methods:**

Fifteen highly trained distance runners (seven male; eight female) completed a single running test composed of 4 × 9-min interval series at fixed percentages of body weight ranging from 0 to 30% body weight support (BWS) in 10% increments on LBPPT. The first interval was always conducted at 0% BWS; thereafter, intervals at 10, 20, and 30% BWS were conducted in random order. Each interval consisted of three stages of 3 min each, at velocities of 14.5, 16.1, and 17.7 km·h^−1^ for men and 12.9, 14.5, and 16.1 km·h^−1^ for women. Expired gases, ventilation, breathing frequency, heart rate (HR), rating of perceived exertion (RPE), and stride characteristics were measured during each running speed and BWS.

**Results:**

Male and female runners had similar physiological and biomechanical responses to running on LBPPT. Increasing BWS increased stride length (*p* < 0.02) and flight duration (*p* < 0.01) and decreased stride rate (*p* < 0.01) and contact time (*p* < 0.01) in small-large magnitudes. There was a large attenuation of oxygen consumption (VO_2_) relative to BWS (*p* < 0.001), while there were trivial-moderate reductions in respiratory exchange ratio, minute ventilation, and respiratory frequency (*p* > 0.05), and small-large effects on HR and RPE (*p* < 0.01). There were trivial-small differences in V_E_, respiratory frequency, HR, and RPE for a given VO_2_ across various BWS (*p* > 0.05).

**Conclusions:**

The results indicate the male and female distance runners have similar physiological and biomechanical responses to LBPPT running. Overall, the biomechanical changes during LBPPT running all contributed to less metabolic cost and corresponding physiological changes.

## Key Points

- Well-trained male and female distance runners have similar physiological and biomechanical responses while running with body weight support on a lower-body positive pressure treadmill.
- When considering the global unweighing effects on stride parameters during running, its major influence was the large increase in flight time, which contrasted the disproportional decrease in contact time resulting in overall longer stride length and reduction in stride rate.
- There was a disproportionate decrease in oxygen consumption relative to body weight support which led to an attenuation of heart rate and rating of perceived exertion and, to a lesser degree, respiratory exchange ratio, minute ventilation, and respiratory frequency between each level of body weight support and running speed.

## Background

**Fig. 1:**
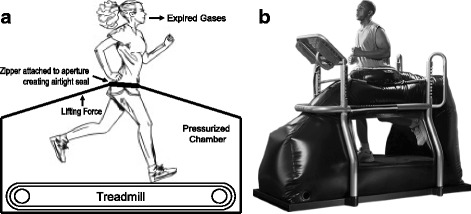
**a** Schematic depictions of the lower-body positive pressure treadmill (AlterG Anti-Gravity Treadmill^®^ P200) used during testing. **b** AlterG Anti-Gravity Treadmill^®^ P200. The lower-body positive pressure treadmill uses an enclosed treadmill body weight support system that makes use of the ability to increase air pressure around the user’s lower body to create a lifting force near the person’s center of mass. The pressurized chamber contains an aperture surrounding the subject’s waist. Each subject wore a pair of flexible neoprene shorts that included a kayak-style spray skirt and a zipper attached to the aperture allowing for an airtight seal around the waist. Expired gases were continuously collected and measured using a metabolic cart, and biomechanical measures were assessed using high-speed video analysis

Body weight support and running velocity both affect the physiological and biomechanical responses of human running [1]. Previous studies show that when running at normal body weight, metabolic demand increases with velocity [2, 3]. The greater metabolic demand of faster running speeds has been attributed to increases in stride frequency, increases in mechanical power, and generation of greater ground reaction forces over shorter periods of ground contact [2, 4, 5]. Coaches and athletes have used the increased metabolic demand associated with faster running velocities as a means of enhancing aerobic capacity and running performance [6, 7]. However, running at fast velocities cannot be sustained over extended durations and greatly increases the risk of overuse and orthopedic injury [8]. Thus, as a way to train at faster running speeds, to add training volume, or when people may not be able to run safely at their full body weight after orthopedic injury and/or surgery, lower-body positive pressure treadmills (LBPPT), such as the AlterG Anti-Gravity Treadmill^®^ (AlterG, Inc., Menlo Park, CA) (Fig. 1) that supports the user’s body weight, have become increasingly commonplace among highly trained athletes [9, 10].

Originally, LBPPT were designed to simulate the musculoskeletal and cardiovascular deconditioning experienced by astronauts during prolonged exposure to microgravity environment [11]. The commercially available LBPPT now have a simple interface that allows the user to select a desired percentage of body weight at which to run. The device then uses small increases in air pressure around the user’s lower body to create a lifting force near the subject’s mass center in order to reduce body weight (Fig. 1) [12]. Compared to the other rehabilitation training options that simulate unloaded running such as harness systems and underwater immersion [13–15], LBPPT are more comfortable and therefore allow for extended usage time. Additionally, LBPPT have relatively less impedance compared to other options, thus simulating normal overground running kinematics and gait patterns [10]. LBPPT also allow for running velocities that exceed the capabilities of even the best distance runners in the world [10, 16], therefore making it an effective training and rehabilitation tool for highly trained and elite athletes. Furthermore, providing body weight support (BWS) allows for attenuation of the biomechanical risks of running so that movements can safely be repeated and improved, potentially allowing athletes to increase training volume or return to running sooner following injury or surgery. However, athletes using LBPPT may experience a cardiovascular fitness decline because of the task’s decreased aerobic demands without modifying the treadmill velocity at which they run [9, 10, 12, 16–24].

Previous research has shown that equivalent maximal and submaximal oxygen consumption (VO_2_) can be achieved while running on LBPPT by increasing the running speed to offset the reduction in oxygen consumption associated with running with BWS [12, 20, 22, 25]. When observed VO_2_ values were compared with predicted values [9, 23, 24], the coefficient of determination (*R* ^2^ = 0.69–0.88) was large to very large, indicating the derived equation in their samples was a good fit and it is feasible to derive similar equations in highly trained populations [1]. All studies that showed reduction in VO_2_ also showed corresponding reductions in heart rate (HR) [9, 12, 18, 22–24, 26, 27]. McNeil et al. [9] also reported decreased respiratory exchange ratio (RER). Raffalt et al. [12] reported decreased minute ventilation (V_E_) during unweighted submaximal running, despite unchanged respiratory rate, whereas Gojanovic et al. [22] reported no change in V_E_. Both Ruckstuhl et al. [18, 27] and McNeil et al. [9] reported decreased rating of perceived exertion (RPE) with unweighted submaximal running, whereas Sainton et al. [26] reported no changes in RPE and Gojanovic et al. [22] reported an increase in RPE during unweighted maximal running. However, rather than comparing changes in RPE across unweighted levels, Hoffman and Donaghe [23] showed that RPE and HR remained the same for a given VO_2_ across various body weight settings. Studies have also found a decrease [22] and no change [12] in blood lactate concentration during unweighted maximal running.

Results are also conflicting regarding stride characteristics while running on LBPPT [10, 12, 22, 26, 28, 29]. Several studies have reported stride rate decreased with unweighting [10, 12, 26, 29], whereas Gojanovic et al. [22] found stride rate increased in males and remained unchanged in females at different absolute velocities. Grabowski and Kram [10] reported an increase in contact time, whereas Raffalt et al. [12] and Neal et al. [29] reported decreased contact time, and Sainton et al. [26] reported no change in contact time. However, studies are in agreement that stride length and flight time increase with unweighting [12, 13, 22, 28, 29]. Sometimes, runners may do a portion of their run on LBPPT and the remainder overground. In studies examining the effects of reloading at 100% body weight after previous unweighting, stride rate decreased in one study [26], whereas it was unchanged in another [10]. Grabowski and Kram [10] found contact time increased, and Sainton et al. [26] found flight time increased following unweighted running, which partially explains the anecdotal heavy-feeling athlete’s experience after prolonged periods on LBPPT.

Unfortunately, while these studies provide valuable insight into the physiological and biomechanical demands of running on LBPPT among healthy and recreational runners, there are conflicting evidence and a paucity of data indicating how these results might apply to highly trained or elite caliber runners at the running speeds they habitually train at, which are considerably faster than those of recreational runners [9]. Of the ten studies that met the inclusion criteria for evaluation of physiologic and stride characteristic parameters in a recent systematic review [1], only two studies included highly trained or elite caliber distance runners [9, 12], neither of which included female runners. Previous research has established that differences in physiological and biomechanical characteristics exist between highly trained male and female runners during submaximal normal body weight running [3, 30]. However, data is limited regarding female athlete’s physiological and biomechanical responses to LBPPT running. Lastly, a comprehensive examination of physiological and biomechanical responses to LBPPT running in the same population is lacking. Therefore, this study was designed to evaluate the physiological and stride characteristic responses to LBPPT running in highly trained male and female distance runners.

## Methods

### Subjects

Fifteen highly trained distance runners (seven male; eight female) participated in this study (Table 1). Not all subjects met the criteria to be classified as “elite” according to Barnes and Kilding [2]; therefore, subjects in the present study were considered “highly trained.” Inclusion criteria were to have 1-mile personal best under 4 min and 10 s or 5 min, 5-km personal best under 14 min and 30 s or 17 min, or 10-km personal best under 30 or 35 min for men and women, respectively. Eight of the subjects (four male; four female) were heel strikers, and seven were mid-forefoot strikers. Foot strike was verified by high-speed video analysis. All subjects had prior experience running on LBPPT and refrained from any activities they were unaccustomed to in the 3 days prior to testing. The study was approved by the Grand Valley State University Human Research Review Committee (Reference No. 14–176-H) and performed in accordance with the standards of ethics outlined in the Declaration of Helsinki. All participants provided informed written consent to participate.

**Table 1:** Subject and training characteristics

	*N*	Age (years)	Body mass (kg)	Height (m)	Training history (years)	Training volume (km·wk^−1^)	IAAF score^a^
Male	7	22.1 ± 0.9	68.4 ± 2.2	1.67 ± 0.07	8.1 ± 1.2	135 ± 21	943 ± 46
Female	8	21.4 ± 2.1	54.3 ± 5.0	1.79 ± 0.03	7.3 ± 2.1	84 ± 19	996 ± 60

Data are mean ± SD

^a^International Association of Athletics Federations (IAAF) 2015 scoring tables [37]

### Experimental Testing

The subjects completed a single running test composed of 4 × 9-min interval series at fixed percentages of body weight ranging from 0 to 30% BWS in 10% increments on the AlterG Anti-Gravity P200 Treadmill^®^ (AlterG^®^, Inc., Fremont, CA) (Fig. 1) set at a 1.0% gradient [31]. Prior to experimental testing, subjects performed a 5-min warmup on the LBPPT (0% BWS) at their own self-selected pace, but below that of the first experimental testing velocity. The first interval was always conducted at 0% BWS; thereafter, intervals at 10, 20, and 30% BWS were conducted in random order. Each interval consisted of three stages of 3 min each (9 min total), at velocities of 14.5, 16.1, and 17.7 km·h^−1^ (4.03, 4.47, and 4.92 m·s^−1^, respectively) for men and 12.9, 14.5, and 16.1 km·h^−1^ (3.58, 4.03, and 4.47 m·s^−1^, respectively) for women, always progressing from the slowest to fastest pace with 10-min recovery between intervals. The treadmill was calibrated before each testing session. The decision to take measurements with 10 to 30% BWS was based on previous research, indicating the actual amount of support provided by LBPPT is the most accurate between 10 and 40% BWS [32], as well as anecdotal observation that 30% BWS tends to be the maximum amount of support prescribed for athletes during training and rehabilitation. Furthermore, we took a capillary blood lactate samples (Lactate Pro, Arkray, KDK, Japan) from the fingertip at the conclusion of each interval and determined blood lactate concentrations remained below 4 mmol·L^−1^ (range 1.9 to 3.4 mmol·L^−1^ at the fastest running velocity), indicating the paces were within normal training paces for these athletes.

Throughout the running test, heart rate (Polar RS800sd, Polar Electro, Kempele, Finland) and expired gases were continuously collected and measured using a metabolic cart (ParvoMedics TrueOne 2400, Salt Lake City, UT) to determine VO_2_, carbon dioxide production (VCO_2_), V_E_, and RER. RPE was determined during each stage using a standard Borg RPE scale upon completion of each interval [33]. Biomechanical measures (stride rate, stride length, contact time, and flight time) were determined using high-speed video analysis (240 frames·s^−1^) while running at each velocity and BWS [30]. The average physiological and biomechanical parameters during the final minute of each running speed and BWS were used for analysis.

### Statistical Analyses

Data analyses were performed using SPSS, version 20.0 (SPSS Inc., Chicago, IL), and customized spreadsheets. Means ± standard deviations were calculated for physiological and biomechanical characteristics using the last minute of data collection for each speed and BWS. Comparisons of the differences between genders were made using a spreadsheet for comparing two groups [34]. The effects of BWS on physiological and biomechanical measures were analyzed with a spreadsheet for post-only crossovers [35]. The value at 0% BWS of the dependent variable was included as a covariate to improve precision of the estimate of the effects. Effects were estimated in percent units via log transformation, and uncertainty in the estimate was expressed as 90% confidence limits. The effect size (ES), which represents the magnitude of the difference between the two conditions in terms of SD, was calculated from the log-transformed data by dividing the change in the mean by the average SD of the two conditions. Magnitudes of effects on all measures were evaluated non-clinically: if the confidence interval overlapped thresholds for small positive and negative values, the effect was deemed unclear; all other effects were reported as the magnitude of the observed value and were evaluated probabilistically as described above, except that threshold values for trivial, small, moderate, large, very large, and extremely large effects were < 0.2, 0.2, 0.6, 1.2, 2.0, and 4.0 of the between-subject standard deviation in the control condition [36]. Linear regression analysis performed on the mean values defined the linear relationship (linear equation and Pearson correlation coefficients) between VO_2_ and running speed.

## Results

Descriptive characteristics of the runners are presented in Table 1. There were trivial differences between men and women in age (*p* = 0.07), training history (*p* = 0.23), and personal bests at racing distances between 1 mile and 10 km as represented by IAAF scores [37] (*p* = 0.82). However, males were moderately taller (*p* = 0.005), very largely heavier (*p* < 0.001), and trained at a very large-amount higher volume than females (*p* = 0.001).

### Biomechanical Responses

Biomechanical responses of male and female athletes to BWS across three running velocities are presented in Tables 2 and 3, respectively. Overall, in both men and women, stride rate and contact time decreased with increasing BWS, while stride length and flight duration increased with increasing levels of BWS. There were small-moderate decreases in stride rate (*p* < 0.01) and moderate-very large decreases in contact time (*p* < 0.01) with increases in BWS for both men and women. Stride length (*p* ≤ 0.02) and flight duration (*p* < 0.01) increased in small-large magnitudes with increasing BWS in both males and females. The largest increases or decreases in biomechanical characteristics tended to occur between 10 and 20% BWS or between 20 and 30% BWS.

**Table 2:** Biomechanical characteristics of male runners by velocity and different levels of body weight support

	14.5 km·h^−1^ (4.03 m·s^−1^)	Difference between 0% BWS ± 90 CL (ES)^a^	Difference between BWS ± 90 CL (ES)^b^	16.1 km·h^−1^ (4.47 m·s^−1^)	Difference between 0% BWS ± 90 CL (ES)	Difference between BWS ± 90 CL (ES)	17.7 km·h^−1^ (4.92 m·s^−1^)	Difference between 0% BWS ± 90 CL (ES)	Difference between BWS ± 90 CL (ES)
Stride length (m)									
0% BWS	1.53 ± 0.07			1.66 ± 0.07			1.75 ± 0.06		
10% BWS	1.57 ± 0.06	2.9 ± 2.3 (0.52)		1.70 ± 0.07	2.7 ± 1.2 (0.53)		1.77 ± 0.06	1.0 ± 0.8 (0.23)	
20% BWS	1.66 ± 0.06	8.5 ± 2.7 (1.46)	5.4 ± 0.8 (0.94)	1.74 ± 0.06	4.8 ± 1.1 (0.94)	2.1 ± 0.9 (0.42)	1.81 ± 0.07	3.3 ± 0.4 (0.76)	2.2 ± 0.9 (0.53)
30% BWS	1.70 ± 0.09	11.1 ± 2.0 (1.89)	2.4 ± 2.1 (0.43)	1.80 ± 0.09	8.5 ± 0.8 (1.63)	3.5 ± 1.5 (0.69)	1.87 ± 0.08	6.4 ± 0.8 (1.48)	3.1 ± 0.7 (0.72)
Stride rate (steps·min^−1^)									
0% BWS	179.7 ± 2.8			181.4 ± 3.0			182.6 ± 2.6		
10% BWS	178.6 ± 2.8	− 0.6 ± 0.4 (0.35)		179.6 ± 2.8	− 1.0 ± 0.4 (0.35)		181.4 ± 3.2	− 0.6 ± 0.4 (0.38)	
20% BWS	175.7 ± 1.8	− 2.2 ± 0.9 (1.24)	− 1.6 ± 0.8 (0.89)	177.9 ± 1.7	− 2.0 ± 1.0 (1.24)	− 0.9 ± 0.8 (0.89)	179.7 ± 2.5	− 1.6 ± 0.5 (0.94)	− 0.9 ± 0.5 (0.56)
30% BWS	172.9 ± 1.4	− 3.8 ± 1.0 (2.14)	− 1.6 ± 0.6 (0.90)	174.8 ± 1.9	− 3.6 ± 1.0 (2.14)	− 1.7 ± 0.5 (0.90)	177.0 ± 2.1	− 3.0 ± 0.8 (1.85)	− 1.5 ± 0.5 (0.91)
Contact time (ms)									
0% BWS	209.4 ± 7.2			197.7 ± 7.2			184.4 ± 6.8		
10% BWS	204.9 ± 8.0	− 2.2 ± 2.0 (0.56)		192.1 ± 7.6	− 2.8 ± 0.9 (0.68)		179.0 ± 6.5	− 2.9 ± 0.7 (0.70)	
20% BWS	197.1 ± 6.9	− 5.9 ± 1.1 (1.52)	− 3.8 ± 1.2 (0.96)	185.6 ± 7.2	− 6.1 ± 1.4 (1.51)	− 3.4 ± 1.8 (0.83)	172.7 ± 7.7	− 6.4 ± 1.0 (1.55)	− 3.5 ± 1.1 (0.85)
30% BWS	185.1 ± 8.7	− 11.6 ± 1.9 (3.12)	− 6.1 ± 1.8 (1.59)	175.9 ± 9.1	− 11.1 ± 2.2 (2.80)	− 5.3 ± 1.6 (1.29)	167.7 ± 7.9	− 9.1 ± 1.2 (2.25)	− 2.9 ± 0.8 (0.69)
Flight duration (ms)									
0% BWS	139.6 ± 12.3			144.6 ± 12.0			155.7 ± 11.6		
10% BWS	147.9 ± 11.8	5.9 ± 1.2 (0.62)		152.4 ± 12.3	5.4 ± 0.8 (0.55)		163.3 ± 10.0	4.9 ± 3.7 (0.56)	
20% BWS	167.4 ± 10.6	20.1 ± 2.5 (1.96)	13.4 ± 1.9 (1.35)	173.1 ± 10.2	19.9 ± 3.6 (1.89)	13.7 ± 3.8 (1.34)	175.4 ± 12.3	12.9 ± 1.3 (1.41)	7.6 ± 3.6 (0.85)
30% BWS	185.9 ± 10.4	33.4 ± 3.0 (3.09)	11.0 ± 1.5 (1.12)	189.7 ± 9.7	31.5 ± 3.9 (2.85)	9.6 ± 1.1 (0.95)	192.7 ± 10.2	23.9 ± 2.3 (2.49)	9.8 ± 1.9 (1.08)

Data are means ± SD

*BWS* body weight support, *CL* confidence limits, *ES* effect size

^a^Represents the difference as percent ± 90 CL and the effects between levels of BWS and 0% BWS (e.g., 0% BWS and 10% BWS; 0% BWS and 20% BWS; 0% BWS and 30% BWS)

^b^Represents the difference as percent ± 90 CL and the effects between 10% BWS and 20% BWS or between 20% BWS and 30% BWS

**Table 3:** Biomechanical characteristics of female runners by velocity and different levels of body weight support

	12.9 km·h^−1^ (4.03 m·s^−1^)	Difference between 0% BWS ± 90 CL (ES)^a^	Difference between BWS ± 90 CL (ES)^b^	14.5 km·h^−1^ (4.47 m·s^−1^)	Difference between 0% BWS ± 90 CL (ES)	Difference between BWS ± 90 CL (ES)	16.1 km·h^−1^ (4.92 m·s^−1^)	Difference between 0% BWS ± 90 CL (ES)	Difference between BWS ± 90 CL (ES)
Stride length (m)									
0% BWS	1.33 ± 0.08			1.55 ± 0.07			1.65 ± 0.08		
10% BWS	1.49 ± 0.07	12.4 ± 1.4 (1.74)		1.60 ± 0.09	3.2 ± 1.5 (0.64)		1.69 ± 0.08	2.3 ± 0.4 (0.45)	
20% BWS	1.52 ± 0.07	14.8 ± 1.6 (2.05)	2.2 ± 0.3 (0.31)	1.65 ± 0.08	6.2 ± 0.9 (1.24)	2.9 ± 1.0 (0.59)	1.73 ± 0.08	4.6 ± 0.5 (0.87)	2.2 ± 0.5 (0.43)
30% BWS	1.62 ± 0.08	22.2 ± 1.7 (2.98)	6.5 ± 0.8 (0.93)	1.71 ± 0.09	10.4 ± 1.0 (2.05)	4.0 ± 0.7 (0.81)	1.77 ± 0.08	7.2 ± 0.5 (1.34)	2.5 ± 0.5 (0.47)
Stride rate (steps·min^−1^)									
0% BWS	182.4 ± 3.3			183.9 ± 3.0			186.1 ± 3.9		
10% BWS	181.6 ± 2.8	− 0.4 ± 0.6 (0.20)		182.9 ± 2.8	− 0.5 ± 0.6 (0.21)		184.5 ± 3.4	− 0.9 ± 0.5 (0.37)	
20% BWS	177.9 ± 2.5	− 2.5 ± 0.7 (1.21)	− 2.1 ± 0.5 (1.01)	179.0 ± 1.7	− 2.6 ± 0.8 (1.20)	− 2.2 ± 0.7 (0.99)	181.8 ± 2.7	− 2.3 ± 0.8 (0.99)	− 1.5 ± 0.5 (0.63)
30% BWS	174.6 ± 2.4	− 4.2 ± 0.9 (2.10)	− 1.8 ± 0.5 (0.89)	176.5 ± 1.9	− 4.0 ± 1.1 (1.83)	− 1.4 ± 0.5 (0.63)	178.6 ± 2.3	− 4.0 ± 1.1 (1.72)	− 1.7 ± 0.6 (0.73)
Contact time (ms)									
0% BWS	224.0 ± 8.2			210.8 ± 6.6			197.1 ± 6.7		
10% BWS	215.6 ± 6.1	− 3.8 ± 1.3 (1.32)		205.1 ± 7.2	− 2.7 ± 0.9 (0.77)		188.3 ± 7.1	− 4.5 ± 1.1 (1.22)	
20% BWS	204.0 ± 6.8	− 8.9 ± 1.9 (3.22)	− 5.4 ± 1.4 (1.90)	187.6 ± 6.2	− 11.0 ± 0.8 (3.31)	− 8.5 ± 0.9 (2.54)	177.8 ± 6.3	− 9.8 ± 0.4 (2.73)	− 5.6 ± 1.3 (1.51)
30% BWS	191.5 ± 8.5	− 14.5 ± 1.4 (5.40)	− 6.1 ± 1.7 (2.18)	180.5 ± 6.3	− 14.4 ± 1.3 (4.42)	− 3.8 ± 0.6 (1.11)	171.1 ± 6.7	− 13.2 ± 0.5 (3.74)	− 3.7 ± 0.5 (1.01)
Flight duration (ms)									
0% BWS	127.5 ± 8.2			130.6 ± 7.7			137.8 ± 7.9		
10% BWS	138.8 ± 6.1	8.9 ± 4.5 (1.15)		143.1 ± 7.1	9.6 ± 2.3 (1.36)		152.8 ± 7.9	10.9 ± 1.0 (1.57)	
20% BWS	160.5 ± 6.8	26.0 ± 2.9 (3.10)	15.7 ± 2.9 (1.64)	169.1 ± 8.8	29.5 ± 1.9 (3.84)	18.1 ± 3.4 (2.48)	173.1 ± 8.0	25.7 ± 1.5 (3.47)	13.4 ± 1.2 (1.90)
30% BWS	181.4 ± 8.5	42.4 ± 2.9 (4.74)	13.0 ± 1.2 (1.96)	185.5 ± 8.0	42.1 ± 2.7 (5.22)	9.7 ± 1.3 (1.38)	190.3 ± 9.1	38.2 ± 1.3 (4.90)	9.9 ± 1.2 (1.43)

Data are means ± SD

*BWS* body weight support, *CL* confidence limits, *ES* effect size

^a^Represents the difference as percent ± 90 CL and the effects between levels of BWS and 0% BWS (e.g., 0% BWS and 10% BWS; 0% BWS and 20% BWS; 0% BWS and 30% BWS)

^b^Represents the difference as percent ± 90 CL and the effects between 10% BWS and 20% BWS or between 20% BWS and 30% BWS

### Physiological Responses

Physiological responses of male and female athletes to BWS across three running velocities are presented in Tables 4 and 5, respectively. There were large-extremely large effects on running economy (VO_2_) at each speed and between all BWS for men (*p* ≤ 0.001) and moderate-large effects for women (*p* < 0.001). There were trivial differences between the first 15 s and the last 15 s of the last minute analyzed at each running speed and BWS, indicating our subjects did achieve a steady state during each interval series. Equations from linear regression analyses defining VO_2_ (ml·kg^−1^·min^−1^) as functions of running speed (m·s^−1^) at each BWS are presented in Table 6. There was increased variability with additional BWS (from 0.993 to 0.973 in men and 0.985 to 0.929 in women); however, coefficients of determinations (*R* ^2^) were nearly perfect between 0 and 30% BWS [36]. For both men and women, effects on RER were trivial-small with only significant differences between 0 and 10% BWS at 16.1 km·h^−1^ (*p* = 0.02) for men and 20 and 30% BWS at 16.1 km·h^−1^ (*p* = 0.04) for women. Minute ventilation and respiratory frequency decreased in trivial-moderate magnitudes with BWS (*p* > 0.05). Heart rate decreased with increasing BWS at moderate-large magnitudes (*p* < 0.01) in men except between 20 and 30% BWS at 16.1 and 17.7 km·h^−1^ (*p* = 0.14 and 0.08, respectively). In females, HR decreased significantly (*p* < 0.05) at 14.5 and 16.1 km·h^−1^ with increasing BWS, but not 12.9 km·h^−1^ (*p* > 0.08). Rating of perceived exertion went down in mostly small-moderate magnitudes with increasing BWS across all speeds (*p* < 0.01). There were trivial-small differences in V_E_, respiratory frequency, HR, and RPE for a given VO_2_ across various BWS (*p* > 0.05).

**Table 4:** Physiological characteristics of male runner by velocity and different levels of body weight support

	14.5 km·h^−1^ (4.03 m·s^−1^)	Difference between 0% BWS ± 90 CL (ES)^a^	Difference between BWS ± 90 CL (ES)^b^	16.1 km·h^−1^ (4.47 m·s^−1^)	Difference between 0% BWS ± 90 CL (ES)	Difference between BWS ± 90 CL (ES)	17.7 km·h^−1^ (4.92 m·s^−1^)	Difference between 0% BWS ± 90 CL (ES)	Difference between BWS ± 90 CL (ES)
Running economy (VO_2_ ml·kg^−1^·min^−1^)									
0% BWS	45.4 ± 2.4			50.6 ± 2.4			57.8 ± 2.9		
10% BWS	39.6 ± 2.7	− 12.8 ± 4.1 (2.29)		44.6 ± 2.1	− 11.9 ± 2.5 (2.39)		49.8 ± 3.4	− 13.9 ± 3.2 (2.57)	
20% BWS	30.9 ± 1.3	− 31.9 ± 4.2 (6.45)	− 21.9 ± 4.0 (4.16)	36.9 ± 1.3	− 27.2 ± 4.8 (6.00)	− 17.4 ± 4.9 (3.61)	41.3 ± 2.6	− 28.6 ± 5.5 (5.78)	− 17.0 ± 6.1 (3.21)
30% BWS	28.1 ± 1.6	− 38.1 ± 7.8 (8.04)	− 9.0 ± 4.6 (1.59)	33.9 ± 1.7	− 33.1 ± 3.5 (7.61)	− 8.1 ± 2.7 (1.60)	37.2 ± 2.5	− 35.8 ± 5.8 (7.61)	− 10.1 ± 1.6 (1.83)
Respiratory exchange ratio (VCO_2_/VO_2_)									
0% BWS	0.82 ± 0.04			0.84 ± 0.03			0.86 ± 0.04		
10% BWS	0.83 ± 0.04	1.9 ± 1.2 (0.29)		0.83 ± 0.03	− 1.2 ± 0.9 (0.24)		0.85 ± 0.03	− 1.6 ± 0.7 (0.28)	
20% BWS	0.83 ± 0.04	0.1 ± 1.4 (0.02)	0.9 ± 1.6 (0.22)	0.83 ± 0.04	− 1.9 ± 1.9 (0.38)	− 1.1 ± 2.5 (0.21)	0.84 ± 0.04	− 1.3 ± 1.3 (0.21)	− 0.1 ± 2.0 (0.01)
30% BWS	0.83 ± 0.04	0.2 ± 1.5 (0.03)	− 1.4 ± 1.4 (0.14)	0.83 ± 0.04	− 2.6 ± 2.4 (0.51)	0.0 ± 1.0 (0.00)	0.84 ± 0.03	− 1.2 ± 2.2 (0.20)	− 0.4 ± 2.4 (0.06)
Minute ventilation (L·min^−1^)									
0% BWS	52.3 ± 5.8			56.8 ± 5.7			60.4 ± 7.8		
10% BWS	50.1 ± 4.6	− 5.2 ± 4.4 (0.38)		53.3 ± 4.6	− 5.7 ± 4.4 (0.51)		59.5 ± 6.0	− 1.3 ± 9.6 (0.09)	
20% BWS	48.6 ± 4.1	− 6.2 ± 5.3 (0.46)	− 4.3 ± 5.6 (0.32)	51.3 ± 6.3	− 9.8 ± 4.3 (0.90)	− 5.5 ± 7.7 (0.50)	55.4 ± 4.8	− 8.0 ± 8.4 (0.56)	− 6.8 ± 6.6 (0.47)
30% BWS	46.2 ± 2.6	− 11.3 ± 10.9 (0.86)	− 3.7 ± 5.4 (0.27)	49.9 ± 4.7	− 12.0 ± 8.8 (1.12)	− 2.5 ± 9.5 (0.22)	54.9 ± 5.0	− 8.7 ± 8.4 (0.61)	− 0.7 ± 2.2 (0.05)
Respiratory frequency (breathes·min^−1^)									
0% BWS	34.4 ± 4.6			35.6 ± 4.7			37.0 ± 5.1		
10% BWS	34.0 ± 5.1	2.2 ± 13.0 (0.12)		34.9 ± 5.0	− 2.2 ± 9.2 (0.13)		36.1 ± 4.6	− 2.8 ± 4.2 (0.20)	
20% BWS	33.4 ± 3.5	− 0.7 ± 10.1 (0.04)	1.3 ± 8.3 (0.07)	32.7 ± 5.7	− 7.8 ± 8.9 (0.49)	− 8.3 ± 9.9 (0.52)	33.8 ± 5.0	− 6.1 ± 4.9 (0.45)	− 6.4 ± 11.6 (0.47)
30% BWS	29.4 ± 4.2	− 12.3 ± 19.0 (0.74)	− 13.0 ± 15.6 (0.78)	31.2 ± 2.7	− 11.3 ± 14.1 (0.71)	− 4.1 ± 16.2 (0.25)	33.6 ± 4.2	− 8.5 ± 9.4 (0.64)	1.0 ± 12.9 (0.07)
Heart rate (BPM)									
0% BWS	143 ± 5			150 ± 6			157 ± 8		
10% BWS	135 ± 6	− 5.0 ± 2.6 (1.33)		143 ± 5	− 4.8 ± 2.7 (0.99)		150 ± 6	− 4.7 ± 2.8 (0.87)	
20% BWS	126 ± 3	− 11.7 ± 3.3 (3.21)	− 7.0 ± 1.9 (1.88)	132 ± 5	− 11.9 ± 3.5 (2.52)	− 7.4 ± 2.1 (1.53)	141 ± 5	− 10.0 ± 3.9 (1.92)	− 5.6 ± 2.0 (1.05)
30% BWS	121 ± 5	− 15.1 ± 4.9 (4.23)	− 3.9 ± 2.2 (1.03)	129 ± 7	− 14.3 ± 3.5 (3.08)	− 2.8 ± 3.2 (0.56)	137 ± 8	− 12.7 ± 4.8 (2.46)	− 2.9 ± 4.1 (0.54)
RPE (6–20 AU)									
0% BWS	13.7 ± 1.3			14.7 ± 1.1			15.6 ± 1.3		
10% BWS	11.9 ± 0.7	− 13.4 ± 6.9 (1.39)		12.6 ± 0.8	− 14.5 ± 4.7 (1.88)		13.6 ± 0.8	− 12.7 ± 6.7 (1.49)	
20% BWS	10.6 ± 0.8	− 22.8 ± 4.8 (2.51)	− 10.9 ± 4.7 (1.12)	11.4 ± 0.8	− 22.3 ± 9.4 (3.03)	− 9.1 ± 5.6 (0.45)	12.4 ± 1.0	− 20.2 ± 9.5 (2.46)	− 8.5 ± 5.5 (0.98)
30% BWS	9.9 ± 0.9	− 28.1 ± 7.4 (3.19)	− 6.9 ± 5.0 (0.69)	11.0 ± 0.6	− 25.2 ± 9.3 (3.48)	− 3.7 ± 4.8 (1.15)	11.7 ± 0.8	− 24.7 ± 10.5 (3.10)	− 5.7 ± 4.1 (0.64)

Data are means ± SD

*AU* arbitrary units, *BPM* beats per minute, *BWS* body weight support, *CL* confidence limits, *ES* effect size, *RPE* rating of perceived exertion, *VO*_*2*_ oxygen consumption

^a^Represents the difference as percent ± 90 CL and the effects between levels of BWS and 0% BWS (e.g., 0% BWS and 10% BWS; 0% BWS and 20% BWS; 0% BWS and 30% BWS)

^b^Represents the difference as percent ± 90 CL and the effects between 10% BWS and 20% BWS or between 20% BWS and 30% BWS

**Table 5:** Physiological characteristics of female runners by velocity and different levels of body weight support

	12.9 km·h^−1^ (3.58 m·s^−1^)	Difference between 0% BWS ± 90 CL (ES)^a^	Difference between BWS ± 90 CL (ES)^b^	14.5 km·h^−1^ (4.03 m·s^−1^)	Difference between 0% BWS ± 90 CL (ES)	Difference between BWS ± 90 CL (ES)	16.1 km·h^−1^ (4.47 m·s^−1^)	Difference between 0% BWS ± 90 CL (ES)	Difference between BWS ± 90 CL (ES)
Running economy (VO_2_ ml·kg^−1^·min^−1^)									
0% BWS	43.5 ± 3.6			48.3 ± 4.0			55.6 ± 4.8		
10% BWS	37.2 ± 4.0	− 14.7 ± 3.5 (1.66)		41.1 ± 5.0	− 15.3 ± 4.4 (1.70)		48.7 ± 5.5	− 13.1 ± 4.1 (2.29)	
20% BWS	29.1 ± 3.3	− 33.2 ± 4.2 (4.22)	− 21.7 ± 2.2 (2.56)	32.0 ± 3.9	− 34.0 ± 5.0 (4.24)	− 22.1 ± 2.5 (2.55)	38.0 ± 4.4	− 31.8 ± 5.1 (6.45)	− 21.6 ± 3.0 (4.16)
30% BWS	26.5 ± 2.9	− 39.2 ± 4.3 (5.21)	− 9.0 ± 2.7 (0.99)	28.7 ± 3.4	− 40.8 ± 5.0 (5.35)	− 10.3 ± 2.5 (1.11)	34.8 ± 4.4	− 37.6 ± 6.4 (8.04)	− 8.5 ± 3.5 (1.59)
Respiratory exchange ratio (VCO_2_/VO_2_)									
0% BWS	0.83 ± 0.06			0.84 ± 0.06			0.86 ± 0.06		
10% BWS	0.82 ± 0.05	− 0.6 ± 6.6 (0.22)		0.81 ± 0.05	− 3.9 ± 5.3 (0.53)		0.84 ± 0.05	− 1.6 ± 6.9 (0.22)	
20% BWS	0.82 ± 0.05	− 0.6 ± 9.0 (0.40)	0.0 ± 2.7 (0.18)	0.83 ± 0.05	− 1.0 ± 7.4 (0.14)	3.0 ± 4.3 (0.39)	0.83 ± 0.04	− 3.0 ± 6.9 (0.40)	− 1.4 ± 2.2 (0.18)
30% BWS	0.81 ± 0.04	− 2.0 ± 8.0 (0.09)	− 1.4 ± 3.6 (0.31)	0.85 ± 0.04	0.4 ± 7.7 (0.06)	1.5 ± 1.8 (0.20)	0.85 ± 0.04	− 0.7 ± 7.6 (0.09)	2.4 ± 2.6 (0.31)
Minute ventilation (L·min^−1^)									
0% BWS	44.7 ± 3.7			50.8 ± 6.0			57.0 ± 7.0		
10% BWS	42.2 ± 5.5	− 6.0 ± 7.3 (0.63)		45.0 ± 6.0	− 11.6 ± 5.8 (0.87)		52.4 ± 6.5	− 8.2 ± 7.3 (0.59)	
20% BWS	38.3 ± 6.8	− 15.2 ± 13.3 (1.69)	− 9.8 ± 7.3 (1.05)	42.3 ± 6.9	− 17.2 ± 6.4 (1.33)	− 6.3 ± 7.7 (0.46)	46.7 ± 6.2	− 18.1 ± 7.9 (1.38)	− 10.8 ± 5.3 (0.79)
30% BWS	36.0 ± 5.3	− 19.9 ± 10.4 (2.28)	− 5.6 ± 8.9 (0.59)	41.1 ± 6.9	− 19.5 ± 7.8 (1.54)	− 2.9 ± 4.6 (0.21)	45.0 ± 7.9	− 21.7 ± 11.1 (1.69)	− 4.4 ± 6.5 (0.31)
Respiratory frequency (breathes·min^−1^)									
0% BWS	38.0 ± 4.6			38.8 ± 4.4			41.2 ± 6.4		
10% BWS	35.9 ± 9.7	− 7.9 ± 21.2 (0.63)		38.3 ± 8.3	− 2.6 ± 14.1 (0.20)		38.7 ± 5.6	− 5.8 ± 16.9 (0.20)	
20% BWS	33.7 ± 4.7	− 11.6 ± 10.6 (0.94)	− 4.0 ± 14.7 (0.31)	34.9 ± 5.4	− 10.4 ± 14.3 (0.84)	− 8.0 ± 13.6 (0.63)	37.1 ± 3.9	− 9.2 ± 14.7 (0.84)	− 3.6 ± 8.4 (0.63)
30% BWS	30.5 ± 4.1	− 19.9 ± 3.8 (1.70)	− 9.4 ± 9.6 (0.76)	32.7 ± 2.9	− 15.7 ± 12.4 (1.30)	− 5.9 ± 8.6 (0.46)	35.8 ± 3.7	− 12.5 ± 17.5 (1.30)	− 3.7 ± 8.4 (0.46)
Heart rate (BPM)									
0% BWS	151 ± 14			161 ± 14			168 ± 14		
10% BWS	142 ± 13	− 5.5 ± 4.3 (0.57)		151 ± 13	− 6.7 ± 4.2 (0.73)		156 ± 15	− 7.4 ± 5.2 (0.88)	
20% BWS	133 ± 16	− 12.0 ± 7.5 (1.27)	− 6.8 ± 7.8 (0.70)	142 ± 15	− 12.1 ± 4.8 (1.37)	− 5.8 ± 4.4 (0.55)	148 ± 13	− 12.2 ± 5.3 (1.49)	− 4.3 ± 5.1 (0.61)
30% BWS	129 ± 18	− 14.7 ± 8.6 (1.58)	− 3.1 ± 13.2 (0.31)	135 ± 15	− 16.6 ± 6.3 (1.92)	− 5.1 ± 9.0 (0.64)	142 ± 15	− 15.9 ± 4.8 (2.00)	− 4.3 ± 5.7 (0.50)
RPE (6–20 AU)									
0% BWS	13.3 ± 1.8			14.4 ± 1.9			15.1 ± 1.7		
10% BWS	11.9 ± 1.6	− 10.4 ± 3.8 (0.70)		13.0 ± 1.5	− 9.5 ± 3.1 (0.66)		13.8 ± 1.6	− 9.2 ± 3.8 (0.73)	
20% BWS	11.0 ± 1.3	− 16.8 ± 5.2 (1.18)	− 7.2 ± 3.2 (0.48)	11.9 ± 1.6	− 17.5 ± 4.1 (1.28)	− 8.9 ± 3.8 (0.62)	12.6 ± 1.7	− 16.7 ± 4.4 (1.39)	− 8.3 ± 3.0 (0.81)
30% BWS	9.8 ± 1.4	− 26.5 ± 6.6 (1.98)	− 11.6 ± 5.3 (0.79)	10.6 ± 1.5	− 26.2 ± 5.2 (2.02)	− 10.6 ± 3.9 (0.74)	11.4 ± 1.6	− 25.1 ± 6.8 (2.19)	− 10.1 ± 8.2 (0.66)

Data are means ± SD

*AU* arbitrary units, *BPM* beats per minute, *BWS* body weight support, *CL* confidence limits, *ES* effect size, *RPE* rating of perceived exertion, *VO*_*2*_ oxygen consumption

^a^Represents the difference as percent ± 90 CL and the effects between levels of BWS and 0% BWS (e.g., 0% BWS and 10% BWS; 0% BWS and 20% BWS; 0% BWS and 30% BWS)

^b^Represents the difference as percent ± 90 CL and the effects between 10% BWS and 20% BWS or between 20% BWS and 30% BWS

**Table 6:** Equations from linear regression analyses defining oxygen consumption (VO_2_ ml·kg^−1^·min^−1^) as functions of running speed (m·s^−1^) at each BWS for male and female runners

% body weight support	Regression equations	*R* ^2^
Male runners		
0% BWS	VO_2_ (ml·kg^−1^·min^−1^) = 12.807 × speed (m·s^−1^) − 10.204	0.993
10% BWS	VO_2_ (ml·kg^−1^·min^−1^) = 10.493 × speed (m·s^−1^) − 5.8871	0.999
20% BWS	VO_2_ (ml·kg^−1^·min^−1^) = 10.706 × speed (m·s^−1^) − 14.521	0.992
30% BWS	VO_2_ (ml·kg^−1^·min^−1^) = 9.2819 × speed (m·s^−1^) − 11.185	0.973
Female runners		
0% BWS	VO_2_ (ml·kg^−1^·min^−1^) = 9.8994 × speed (m·s^−1^) − 4.2691	0.985
10% BWS	VO_2_ (ml·kg^−1^·min^−1^) = 9.336 × speed (m·s^−1^) − 6.6136	0.967
20% BWS	VO_2_ (ml·kg^−1^·min^−1^) = 7.3159 × speed (m·s^−1^) − 5.2166	0.957
30% BWS	VO_2_ (ml·kg^−1^·min^−1^) = 6.8762 × speed (m·s^−1^) − 5.6689	0.929

*BWS* body weight support, *VO*_*2*_ oxygen consumption, *R* ^*2*^ proportion of explained variability

## Discussion

This is the first study to assess the physiological and biomechanical responses of running on a LBPPT among highly trained female distance runners across typical training speeds and different levels of BWS, while it adds to the body of literature regarding highly trained male distance runners. The most important finding of the current study is that male and female runners had similar physiological and biomechanical responses to running on LBPPT. In general, we found that increasing BWS on a LBPPT increased stride length and flight duration and decreased stride rate and contact time while attenuating a variety of physiological characteristics at a range of treadmill velocities.

Biomechanical changes to LBPPT running are not well defined and, in the case of stride characteristics, are inconsistent. When considering the global unweighing effects on stride parameters during running, its major influence was the large increase in flight time, which contrasted the disproportional decrease in contact time resulting in overall longer stride length and reduction in stride rate. Sainton et al. [26] described this pattern of movement as similar to the gait patterns adopted by astronauts on the Moon as defined by Minetti et al. [38]. Studies are in agreement that unweighing-induced changes in flight time increase with increasing levels of BWS [12, 13, 26, 29]. In our study, flight time was less affected by higher running speeds but was significantly affected by BWS. Presumably, the BWS provides a longer swing phase and theoretically lowers the working demand of the hip flexion muscles [12].

Both the current data and those of previous studies [12, 22, 28, 29] are in disagreement with the work of Mercer and Chona [39] who reported no effects on stride length with increased BWS. We found small to moderate increases in stride length with each 10% incremental increase in BWS. These findings support our observation that flight time increases with BWS, in line with others’ observations of increased flight times and stride durations [12, 13, 26, 29]. The effect of BWS on stride length seems to be less when running at faster speeds. There was a 22% increase in stride length at 12.9 km·h^−1^ from 0 to 30% BWS compared to only 7% increase at 16.1 km·h^−1^ in females. Raffalt et al. [12] reported similar phenomena in stride rate and stride length. In this study, flight time was also affected less at faster running velocities, indicating that the running pattern was less affected by BWS at higher running speeds.

There is a natural reciprocal relationship between stride length and stride rate [30]; therefore, it is not surprising then to find reductions in stride rate, considering the increases in stride length in the present study and others with increasing BWS [10, 12, 26, 29]. Only Gojanovic et al. [22, 25] have reported increases in stride rate during BWS running, but that was only in male runners during maximal or supramaximal running velocities. Mercer and Chona [39] and Gojanovic et al. [22] reported no change in stride rate with BWS in males and females during maximal or supramaximal running speeds. An increase in stride rate has been reported to reduce the risk of chronic knee injuries by reducing joint loading and increasing gluteal activation [40]. Conversely, decreasing stride rate may place the knee joint under greater load and potentially increase the risk of injury [29]. Given that LBPPT are intended for rehabilitating injured athletes, preventing injuries, and enhancing performance, it is important that training benefits achieved on LBPPT translate to overground running too [1]. Our results and others support that running on a LBPPT at submaximal running speeds promotes a decrease in stride rate [10, 12, 26, 29], which may not be beneficial for athletes trying to add training volume, prevent injury, or rehabilitate an injury. However, there is no evidence suggesting the effects of LBPPT running persist for a long term after returning to overground running.

In contrast to Grabowski and Kram [10] and Farley and McMahon [41] who reported an increase in contact time and Sainton et al. [26] who found no change in contact time, we found significant decreases in contact time with increased BWS across all speeds. Several other studies also found decreases in contact time with BWS [12, 29]. Sainton et al. [26] suggest that this is a result of greater knee extension and ankle plantar flexion while running on a LBPPT because the aperture (Fig. 1) provides progressively more lifting force with increased BWS. Furthermore, as BWS is increased, a shift in regional loading of the shoe towards the forefoot occurs due to this lifting force that may result in reduced contact time and altered running patterns, particularly at ≥ 20% BWS [42]. The study of Raffalt et al. [12] is one of the few other studies using high-trained runners and suggests this could simply be explained by the participant’s better ability to apply force to the treadmill belt due to their experience with LBPPT running and training background. However, running at the same speed with increased BWS has also been shown to decrease the vertical ground reaction force impulse [13, 15] which can be achieved either by lower peak vertical ground reaction forces or by shorter contact times.

The conflicting findings in biomechanical responses, particularly contact time, between studies may depend on a number of factors, including the magnitude of unloading (e.g., 20% BWS may produce different results than 60% BWS), accuracy of unloading, running speed, fitness levels of subjects, treadmill running experience, and more specifically, LBPPT running experience [1, 32]. According to a recent systematic review, the current LBPPT literature suggests that there are non-linear changes in muscle activity with different increments of BWS [1]. Some data indicate certain muscles are not affected until significant increases in BWS occur (e.g., significant differences present at 40% BWS but not 20% BWS) [26]. However, other data suggest some muscles experience changes with minimal BWS (i.e., 10% BWS) but not with further unweighting (i.e., 30% BWS), while some muscle experience consistent changes with incremental increases in BWS [39, 43]. Additionally, there is evidence of an accommodation effect, such that previous research has shown multiple trials of unweighted running are necessary for reliable measures of metabolic cost to be made [21], and this may be the case with biomechanical characteristics too.

The biomechanical changes in the present study with LBPPT running contribute to less metabolic cost and corresponding physiological changes. Specifically, if less force needs to be produced to support body weight, neuromuscular activation is reduced, and therefore, there is a decreased need for energy production [1, 2]. Accordingly, it is not surprising that, as BWS increased within a given running velocity, running economy was improved (metabolic demand is decreased), and thus, HR, respiratory frequency, and minute ventilation were also reduced. Our results support a growing body of literature that metabolic cost is reduced with BWS [9, 10, 12, 18, 20–23, 26]. In the present study, we saw very large to extremely large reductions in VO_2_ from 0 to 10% BWS and from 10 to 20% BWS; however, the effect, while still moderate to large in magnitude, was attenuated with additional BWS between 20 and 30% BWS. Kline et al. [20] reported that a proportion of metabolic demand to BWS was nearly equivalent between 10 and 30% BWS; however, with additional BWS, the proportion of metabolic demand differed significantly from the proportion of BWS. Here, the proportional reduction in metabolic cost was greater than that of BWS, particularly between 10 and 20% BWS and between 20 and 30% BWS. Fortunately, previous research has shown that equivalent maximal and submaximal VO_2_ can be achieved while running on LBPPT by increasing the running speed to offset the reduction in oxygen consumption associated with running with BWS [12, 20, 22, 25].

It is important to note that the metabolic demands of running at 0% BWS on a LBPPT are lower than those of running on a regular treadmill [32]. McNeill et al. [9] showed that standing weight on a LBPPT was on average 7% lower than predicted body weight which according to Kline et al. [20] resulted in a 3–9% lower metabolic cost at running velocities between 8.0 and 17.7 km·h^−1^ [32]. The reductions in weight and metabolic demand have been attributed to the inflation of the chamber, which provides additional vertical and horizontal support in the mediolateral direction and reduces the need to propel in the anterior-posterior direction [1, 32, 44]. Thus, in the present study, the proportional decreases in metabolic demand are likely greater than that presented when compared to regular treadmill running. Similar to previous research reporting increased variability in metabolic demand with increased BWS [9], we also found increased variability (from 0.999 down to 0.929) from 0 and 30% BWS; however, our coefficients of determinations (*R* ^2^) were substantially higher than those of previous studies [9, 23, 24]. The differences could be attributed to differences in methodology between studies. Multiple studies have implemented multiple day testing protocols to assess the effects of LBPPT running [9, 10, 12, 20, 21, 23] which may be warranted during long duration or maximal running protocols where the slow drift of VO_2_ may be present. However, Barnes and Kilding [2] reported that the intraindividual variation (typical error) in metabolic cost is attributable to a number of factors and is between 1.3 and 5% at speeds between 12 and 18 km·h^−1^ in well-trained and elite caliber athletes. Thus, we utilized a randomized single-session approach, thus reducing the day-to-day variability possibly seen in other studies. Additionally, our subjects were highly trained distance runners who were used to running 12–20 km during a typical training session; thus, the demand to run 36 min on a LBPPT is relatively negligible to their typical training regimen. This is supported by the fact that RER values did not exceed 1.0 and blood lactate samples at the conclusion of each 9-min interval series were < 4 mmol·L^−1^ (range 1.9 to 3.4 mmol·L^−1^ at the fastest running velocity). Only one other study has reported changes in RER during submaximal LBPPT running [9]. Our results agree with McNeill et al. [9] in that there were no significant effects on RER with BWS at slower running velocities; however, at faster speeds, a significant reduction occurred in both men and women. It should be noted, however, that the 3-min intervals used in this study represent a relatively short time period to reach a physiological steady state and, in a lesser-trained population, would not be appropriate for analysis. However, our data indicated there was no difference between the first 15 s and the last 15 s of the last minute analyzed, indicating our subjects did achieve a steady state.

In addition to the reduction in oxygen consumption, we also found a significant decrease in HR and RPE during unweighted running. Studies are in agreement that there are concomitant decreases in HR and VO_2_ during BWS running [9, 12, 18, 22, 23, 26, 28, 45]. Changes in HR tended to be similar across running speeds; however, the magnitude of effects was attenuated with increases in BWS. Gojanovic et al. [22] suggest that the decrease in HR might be linked to a positive effect of lower-body positive pressure on venous return, which, in turn, might be compensated adequately by an increase in stroke volume. However, we observed that HR was similar at that same VO_2_ independent of BWS, indicating that LBPP on its own did not affect blood flow, stroke volume, and subsequently, HR. In contrast to several studies that reported no significant change in RPE with BWS [23, 26, 45], the reduction in RPE in our study agrees with McNeill et al. [9] and Ruckstuhl et al. [18] who also reported significant reductions in perception of effort at fixed running speeds with increasing BWS. The findings of this study and other studies suggest that BWS does not alter the relationship between HR and VO_2_ or RPE and VO_2_, which indicates that using HR or RPE to prescribe training intensities does not require adjustment when running on a LBPPT with BWS [9, 18, 23].

In addition to examining changes in physiological characteristics across levels of BWS, we found that when compared at the same VO_2_ across various BWS, minute ventilation, respiratory frequency, HR, and RPE were similar. Hoffman and Donaghe [23] also showed that HR and RPE remained the same for a given VO_2_ (25 ml·kg^−1^·min^−1^) across various BWS in healthy untrained subjects. Because the speed necessary to achieve a given VO_2_ must be increased with BWS [12, 20, 22, 25], the unaltered relationship between RPE and VO_2_ suggests that the stimulus for perceived effort is related more to aerobic demand than actual running speed [23]. The other physiological responses (HR, ventilation, respiratory frequency) are consistent with other studies describing physiologic correlates with steady-state oxygen consumption at specific running velocities [2, 3].

## Conclusions

In conclusion, our results indicate the male and female distance runners have similar physiological and biomechanical responses to LBPPT running. When considering the overall effects of BWS on stride parameters, its major influence was the large increase in flight time, which contrasted the smaller decrease in contact time resulting in overall longer stride length and reduction in stride rate. There was a disproportionate decrease in oxygen consumption relative to BWS which led to an attenuation of all other physiological characteristics measured to varying magnitudes between each level of BWS and running speed. The rationale for the disproportional decrease in VO_2_ has not been fully elucidated but may be related to biomechanical changes leading to reduced neuromuscular activation at a given running velocity and, therefore, a decreased need for energy production resulting in less metabolic cost and corresponding physiological changes.

## Abbreviations

AU: Arbitrary units
BPM: Beats per minute
BWS: Body weight support
CL: Confidence limits
ES: Effect size
HR: Heart rate
IAAF: International Association of Athletics Federations
LBPPT: Lower-body positive pressure treadmill
RER: Respiratory exchange ratio
RPE: Rating of perceived exertion
V_E_: Minute ventilation
VO_2_: Oxygen consumption
